# Dyslipidemias and cardiovascular risk scores in urban and rural populations in north-western Tanzania and southern Uganda

**DOI:** 10.1371/journal.pone.0223189

**Published:** 2019-12-06

**Authors:** Bazil Kavishe, Fiona Vanobberghen, David Katende, Saidi Kapiga, Paula Munderi, Kathy Baisley, Samuel Biraro, Neema Mosha, Gerald Mutungi, Janneth Mghamba, Peter Hughes, Liam Smeeth, Heiner Grosskurth, Robert Peck

**Affiliations:** 1 Mwanza Intervention Trials Unit, National Institute for Medical Research, Mwanza, Tanzania; 2 MRC Tropical Epidemiology Group, London School of Hygiene & Tropical Medicine, London, United Kingdom; 3 Medical Research Council/Uganda Virus Research Institute and London School of Hygiene and Tropical Medicine Research Unit, Entebbe, Uganda; 4 Ministry of Health, Kampala, Uganda; 5 Ministry of Health Community Development Gender Elderly and Children, Dar es Salaam, Tanzania; 6 Weill Bugando School of Medicine, Mwanza, Tanzania; 7 Weill Cornell Medical College, New York, NY, United States of America; University of the Witwatersrand, SOUTH AFRICA

## Abstract

**Background:**

Dyslipidemia is a leading risk factor for atherosclerotic cardiovascular disease. There are few published epidemiological data regarding dyslipidemia in Africa. We determined full lipid and apolipoprotein profiles and investigated factors associated with lipid levels in urban and rural populations of north-western Tanzania and southern Uganda.

**Methods:**

We conducted a cross-sectional survey of randomly-selected, community-dwelling adults (≥18yrs) including five strata per country: one municipality, two district towns and two rural areas. Participants were interviewed and examined using the World Health Organization STEPwise survey questionnaire. Serum levels of total cholesterol, low-density lipoprotein cholesterol, high-density lipoprotein cholesterol, triglycerides, and apolipoproteins were measured. Factors associated with mean lipid levels were assessed by multivariable linear regression. Framingham 10-year cardiovascular risk scores were calculated with and without lipids.

**Results:**

One-third of adults in the study population had dyslipidemia. Low high-density lipoprotein cholesterol affected 32–45% of rural adults. High total cholesterol, low-density lipoprotein cholesterol, and apolipoprotein B were found in <15% of adult population in all strata, but were more common in urban adults. Factors independently associated with higher mean low-density lipoprotein cholesterol and apolipoprotein B were female gender, older age, higher education, higher income, obesity, and hypertension. Framingham cardiovascular risk scores with and without lipids yielded similar results and 90% of study subjects in all strata were classified as “low risk”. Among older adults (>55 years), 30% were classified as “high” or “very high” risk.

**Conclusions:**

Dyslipidemias are common among adults in north-western Tanzania and southern Uganda affecting one third of adult population. Overall, cardiovascular risk scores are low but high risk scores are common with older adults. Health services designed and equipped to diagnose and treat dyslipidemia are urgently needed.

## Introduction

Dyslipidemia is a major modifiable risk factor for morbidity and mortality worldwide, particularly as a cause of ischemic heart disease and stroke [1, 2]. According to the most recent global burden of diseases study, there were 4.3 million deaths in 2015 that were attributable to dyslipidemia, making this the 7^th^ leading risk factor for disability-adjusted life years [3]. Although dyslipidemia was traditionally considered a “disease of affluence”, there is a growing recognition that dyslipidemia is also common in low-income regions such as sub-Saharan Africa (SSA) [1, 4, 5].

Little is known about the epidemiology of dyslipidemia in SSA. Findings from few published hospital-based studies [1, 5, 6] may not be representative of the general population. Although several community-based studies on dyslipidemia have been published from SSA [7–16], none of these have included more than one country and few have included both urban and rural areas [11–13]. Moreover, few studies conducted in this region measured full lipid profile including apolipoproteins [14–16].

To contribute data which may help to describe the patterns of dyslipidemia and cardiovascular risk in SSA, we conducted this study to determine full lipid and apolipoprotein profiles and investigate factors associated with lipid levels in urban and rural populations of north-western Tanzania and southern Uganda. We also determined 10-year cardiovascular risk according to the Framingham risk score, with and without lipids. Results from this study may help to guide public health efforts for prevention of cardiovascular diseases in SSA.

## Materials and methods

### Study design and sampling

We conducted a cross-sectional population-based survey among adults aged ≥18 years in north-western Tanzania and southern Uganda from May 2012 to April 2013. As previously described [17], we used stratified, multistage sampling, including five strata per country: a municipal area (Mwanza city in Tanzania; Entebbe town in Uganda), two district towns (Geita and Kahama in Tanzania; Wakiso and Mpigi in Uganda) and two rural areas corresponding to the respective district towns.

### Description of the study area

Mwanza is the second largest commercial city in Tanzania with a population of approximately 710,000 [18]. Entebbe is mainly an administrative town with a population of approximately 70,000 [19]. Kahama, Geita and Mpigi districts are predominantly rural, with a population of approximately 960,000 in Geita, 770,000 in Kahama and 250,000 in Mpigi [18, 19]. Wakiso district is comprised of urban, peri-urban and rural areas and has a population of around 2 million. Mwanza and Entebbe municipalities’ economy depends mainly on medium to large income businesses. The main economic activities in the district towns and rural areas are small entrepreneurship, subsistence farming and fishing.

### Sample size

As previously described [17], the survey was designed to measure the prevalence of selected chronic diseases for intervention planning. We visited 540 randomly selected households to enroll the target sample size of 950 participants per country.

### Study procedures

Data collection was conducted by trained clinicians and nurses at participants’ homes or nearby communal places. Participants were interviewed using a structured questionnaire adapted from the World Health Organization (WHO) STEPwise approach to chronic disease risk factor surveillance (STEPS) instrument [20]. We collected information on socio-demographic characteristics and risk factors for non communicable diseases, including alcohol consumption using a standardized screening questionnaire (Alcohol Use Disorders Identification Test, AUDIT) [21]. We conducted a physical examination to determine weight, height, body mass index (BMI) and blood pressure (BP) as previously described [17]. In summary, weight was measured using digital seca® 813 scale and height using a seca® 213 stadiometer. BP was measured using the Omron digital automatic blood pressure monitor model M6 (Omron Health Care Manufacturing Vietnam Co., Ltd, Binh Duong Province, Vietnam).

During the first visit, we collected non-fasting blood samples for determination of serum levels of total cholesterol (TC), low-density lipoprotein cholesterol (LDL-C), high-density lipoprotein cholesterol (HDL-C), triglycerides, apolipoprotein A1 (apoA1) and apolipoprotein B (apoB) and for HIV and random blood glucose (RBG) tests. Participants found with elevated RBG (≥7mmol/l) were given instructions to fast overnight and a follow up visit was arranged to collect fasting blood glucose (FBG) samples.

In Tanzania, serum aliquots for lipid testing were stored in a portable freezer (-20°C) in the field and transported within five days to the National Institute for Medical Research laboratory in Mwanza municipality where they were stored at -80°C. About two months later, serum samples from Tanzania were transported to the Medical Research Council/Uganda Virus Research Institute (MRC/UVRI) and London School of Hygiene and Tropical Medicine laboratory in Entebbe, Uganda for testing. In Uganda, serum aliquots from the field were transported within 10 hours in a cold box to the MRC/UVRI and London School of Hygiene and Tropical Medicine laboratory where they were immediately stored at -80°C until testing.

### Laboratory methods

Serum lipid and apolipoprotein levels were measured using the Cobas Integra 400 *Plus* analyzer (Roche, Basel, Switzerland) with Roche ‘closed’ reagents (Roche, Manheim, Germany). HDL-C and LDL-C were each directly measured with homogeneous colorimetric assays using HDL-C plus 3^rd^ generation and LDL-C plus 2^nd^ generation reagents. Triglycerides and TC were measured using an enzymatic colorimetric assay (triglyceride and TC generation 2 reagents), and apoA1 and apoB levels by an immunoturbidimetric assay (Tina-quant version 2 reagents). External quality assurance for apoA and apoB was conducted by the College of American Pathologists. The MRC/UVRI and London School of Hygiene and Tropical Medicine laboratory, where all lipid assays were performed, has been certified for good clinical laboratory practice since 2009.

As previously reported [17] we tested venous blood samples in the field for RBG using Accu-Check® Aviva (Roche Diagnostics GmbH, Mannheim, Germany) and FBG using HemoCue® Glucose 201 RT (HemoCue AB, Ängelholm, Sweden).

### Definitions

We defined abnormal lipid levels based on the Adult Treatment Panel III criteria [22] as follows: high TC (≥6.2 mmol/L), high LDL-C (≥4.1 mmol/L), low HDL-C (<1 mmol/L) and high triglycerides (≥2.26 mmol/L). High TC/HDL-C ratio was defined as >6.4 for men and >5.6 for women [23]. High apoB (females >4.43 Umol/L and males >5.04 Umol/L) and low apoA1 (females <1.08 Umol/L and males < 1.04 Umol/L) were defined according to the package insert [24, 25]. Dyslipidaemia was defined as abnormal TC, LDL-C, HDL-C, triglyceride and/or apoB levels.

Hypertension was defined as systolic BP ≥140 mmHg and/or diastolic BP ≥90 mmHg, or currently taking medication for hypertension. Diabetes mellitus was defined as RBG >11.1 mmol/L, or FBG ≥7 mmol/L, or being on diabetes medication. HIV infection was diagnosed based on the national testing algorithms as previously described [17]. BMI (kg/m^2^) was classified as underweight (<18.5), normal weight (18.5-<25), overweight (25-<30) and obese (≥30). Abdominal obesity was defined as waist circumference >94 cm for men and >80 cm for women [26].

The 10-year cardiovascular risks were calculated using the Framingham risk calculator as recommended by current WHO and South African guidelines [27, 28]. We used two versions of this Framingham risk calculator for the sake of comparison, one with lipid values and one without [29]. Age, sex, diabetes, smoking, systolic BP and whether or not on hypertensive treatment are used in both calculations. TC and HDL-C were used in the calculation with lipid results; BMI was used in the calculation without lipid results. Cardiovascular risk scores were categorized as low risk (<10%) intermediate risk (10–19%), and high risk (≥20%) as recommended by the WHO [28].

### Statistical analysis

Data were captured using Ultra Mobile Personal Computers in Uganda and paper-based questionnaires in Tanzania as described previously [17]. Analyses were conducted using Stata Version 11 (Stata Corporation, College Station, USA). We used Stata survey procedures for all analyses to account for the complex sampling design, with sampling weights to account for differential probability of selection within strata.

We included participants for whom we had any lipids data. We tabulated the characteristics of the survey population, prevalence of dyslipidemias and Framingham 10-year cardiovascular risk scores stratified by country and location (municipalities, district towns, and rural areas), using weighted percentages and 95% confidence intervals (CI), and observed (i.e. unweighted) frequencies. Blood lipid distributions were summarized similarly using weighted means and 95% CI. Framingham 10-year cardiovascular risk scores were also analyzed stratified by age groups.

We investigated factors associated with higher LDL-C levels combining data from both countries and using linear regression. Potential determinants of LDL-C were examined using a conceptual framework [30], with four levels (sociodemographic, behavioural, anthropometric and chronic disease factors). Stratum, age and sex were considered *a priori* confounders so were included in all models, therefore comparisons were essentially within the (approximately self-weighted) strata and sampling weights were not applied. Socio-demographic factors were added to the stratum, age and sex-adjusted models and retained if associated with LDL-C at p<0.1. Behavioural factors were then added one by one and retained if they remained associated at p<0.1. Associations with anthropometric and chronic disease factors were subsequently determined in a similar way. This strategy allowed us to assess the effects of variables at each level of the framework, adjusted for variables that are more distal. In reporting, we focus on variables with associations that were statistically significant at p-values of <0.05. Similar analyses were performed for HDL-C, apoA1 and apoB. We performed a sensitivity analysis to investigate factors associated with abnormally high LDL-C, high apoB and low HDL-C using logistic regression. This analysis yielded results similar to those from the linear regressions. We also determined the correlation between apoA1 and HDL-C levels as well as apoB and LDL-C levels using Pearson’s correlation coefficients.

### Ethical considerations

The population survey was approved by the Ugandan National Council for Science and Technology and by the ethics committees of the Tanzanian National Institute for Medical Research, the UVRI, and the London School of Hygiene and Tropical Medicine. We obtained written informed consent (witnessed for illiterate participants) from all participants before administering study procedures. Participants diagnosed with any medical condition during the survey were referred to the nearest public health facility for follow-up care. Those diagnosed with dyslipidemia or high Framingham 10-year cardiovascular risk scores were not referred because lipid-lowering drugs were then not available in the health care system for both countries.

## Results

Lipids data were missing for 52 Tanzania and 2 Uganda participants, leaving 1043 Tanzania and 914 Uganda participants included in the analysis. Of the 54 total participants with missing data: 49 refused blood collection, venipuncture failed in 4 and 1 could not complete study procedures due to a personal emergency.

### Population characteristics

Population characteristics are presented in Table 1, by location (municipal, district town, rural) and country, and these have been described in detail previously [17]. The median age ranged from 28 to 35 years and was highest in rural areas in both Tanzania and Uganda. The study population comprised more women (52–62%) than men across all strata. The prevalence of overweight or obesity, as determined by BMI, was 28–35% in Uganda and 11–27% in Tanzania and was lower in rural areas.

**Table 1:** Characteristics of the study population.

	Weighted %^1^ (95% CI)^2^	Unweighted n^3^	Weighted %^1^ (95% CI)^2^	Unweighted n^3^	Weighted %^1^ (95% CI)^2^	Unweighted n^3^
**TANZANIA**	**Mwanza municipality (n = 170)**		**District towns (n = 326)**		**Rural areas (n = 547)**	
Age						
Weighted median (IQR)^4^	29 (22–41)	170	28 (23–37)	326	34 (24–49)	547
Sex						
Male	42.5% (34.8–50.7)	72	44.6% (38.1–51.3)	145	47.8% (43.7–51.9)	260
Female	57.5% (49.3–65.2)	98	55.4% (48.7–61.9)	181	52.2% (48.1–56.3)	287
Level of education						
None/incomplete primary	21.8% (14.9–30.8)	34	21.0% (15.7–27.4)	70	48.2% (40.6–55.9)	257
Primary	47.9% (43.3–52.5)	82	50.6% (45.4–55.8)	163	43.7% (37.9–49.7)	246
Secondary or above	30.3% (24.7–36.6)	54	28.4% (21.5–36.5)	93	8.1% (5.2–12.4)	44
Smoking status						
Current	8.7% (3.6–19.7)	15	7.7% (4.0–14.6)	26	12.3% (9.4–16.0)	67
Ex-smoker	6.0% (2.3–14.6)	9	8.3% (4.8–13.9)	25	7.9% (5.8–10.6)	45
Never	85.3% (73.2–92.5)	146	84.0% (78.2–88.4)	275	79.8% (76.3–82.9)	435
Alcohol consumption^5^						
Never drinker	68.4% (59.4–76.1)	118	58.4% (51.3–65.2)	192	65.7% (59.3–71.6)	359
Ex-drinker	12.3% (8.8–16.9)	21	25.7% (20.6–31.4)	80	24.0% (19.6–29.2)	136
Healthy drinker^6^	3.9% (2.1–7.2)	7	4.4% (2.5–7.7)	14	4.8% (2.3–9.7)	21
Unhealthy drinker	15.5% (11.1–21.2)	24	11.5% (7.8–16.7)	38	5.5% (3.3–8.9)	31
Eats at least 1 serving of fruit/vegetable per day^7^						
No	28.5% (19.2–40.1)	42	25.2% (17.6–34.7)	63	37.3% (27.6–48.2)	145
Yes	71.5% (59.9–80.8)	104	74.8% (65.3–82.4)	185	62.7% (51.8–72.4)	251
Body Mass Index (BMI) category (kg/m^2^)^8^						
Under weight (<18.5)	13.0% (9.8–17.0)	22	6.9% (4.0–11.8)	22	12.7% (9.9–16.1)	65
Normal weight (18.5 - <25)	61.5% (55.5–67.2)	105	66.3% (59.3–72.6)	218	76.0% (71.1–80.4)	414
Overweight (25 - <30)	17.8% (13.8–22.7)	29	13.3% (10.7–16.3)	43	8.8% (6.2–12.3)	51
Obese (≥30)	7.7% (5.3–11.0)	13	13.6% (9.1–19.8)	43	2.5% (1.3–4.8)	13
Abdominal obesity^9^	28.9% (23.5–34.9)	47	31.7% (24.0–40.5)	102	19.4% (15.4–24.2)	110
**UGANDA**	**Entebbe municipality (n = 205)**		**District towns (n = 278)**		**Rural areas (n = 431)**	
Age						
Weighted median (IQR)^4^	29 (22–39)	205	28 (22–37)	278	35 (24–49)	431
Sex						
Male	38.5% (33.9–43.4)	79	41.0% (34.4–48.1)	108	42.6% (37.5–48.0)	188
Female	61.5% (56.6–66.1)	126	59.0% (51.9–65.6)	170	57.4% (52.0–62.5)	243
Level of education						
None/incomplete primary	17.6% (9.8–29.5)	36	22.2% (18.4–26.4)	67	42.4% (32.7–52.8)	198
Primary	12.2% (6.8–20.8)	25	11.7% (6.3–20.6)	34	18.5% (15.5–22.1)	83
Secondary or above	70.2% (59.3–79.3)	144	66.2% (57.5–73.8)	177	39.0% (30.4–48.4)	150
Smoking status^10^						
Current	7.8% (3.6–16.0)	16	6.9% (4.4–10.6)	15	8.7% (5.3–13.9)	39
Ex-smoker	8.3% (5.0–13.4)	17	5.2% (3.1–8.4)	14	9.2% (7.1–11.9)	39
Never	83.9% (73.7–90.6)	172	87.9% (83.2–91.5)	248	82.1% (75.4–87.3)	353
Alcohol consumption						
Never drinker	32.2% (26.2–38.8)	66	45.6% (38.4–52.9)	121	38.4% (31.5–45.8)	166
Ex-drinker	23.4% (15.2–34.3)	48	22.6% (18.2–27.7)	63	23.5% (18.0–30.1)	103
Healthy drinker^6^	33.7% (27.5–40.4)	69	25.7% (20.2–32.0)	83	33.5% (25.2–43.1)	142
Unhealthy drinker	10.7% (5.9–18.7)	22	6.2% (3.9–9.7)	11	4.5% (2.7–7.5)	20
Eats at least 1 serving of fruit/vegetable per day						
No	44.9% (34.6–55.6)	92	58.8% (51.9–65.5)	182	66.4% (60.1–72.2)	293
Yes	55.1% (44.4–65.4)	113	41.2% (34.5–48.1)	96	33.6% (27.8–39.9)	138
Body Mass Index (BMI) category (kg/m^2^)^11^						
Under weight (<18.5)	2.0% (0.7–5.7)	4	4.1% (1.5–10.6)	16	12.2% (8.6–17.2)	56
Normal (18.5 - <25)	67.0% (58.7–74.4)	134	61.3% (54.7–67.6)	172	60.1% (53.2–66.7)	266
Overweight (25 - <30)	19.0% (14.1–25.1)	38	22.9% (18.2–28.4)	60	20.1% (16.5–24.3)	79
Obese (≥30)	12.0% (7.4–18.8)	24	11.6% (8.0–16.6)	27	7.5% (5.3–10.6)	29
Abdominal obesity^9^^,^^12^	27.5% (19.9–36.6)	56	31.4% (23.4–40.7)	87	27.4% (22.9–32.6)	109

^1^Weighted estimates, adjusted for survey design with sampling weights applied because the probability of being sampled differed within the strata.

^2^95% confidence interval.

^3^Actual number of participants without sampling weights applied.

^4^Interquartile range.

^5^Alcohol consumption results are missing for 2 district town participants.

^6^Not more than 2 alcoholic drinks for female or not more than 3 alcoholic drinks for male per day.

^7^Fruit and vegetable intake results are missing for 24 Mwanza municipality, 78 district town and 151 rural participants.

^8^BMI category results are missing for 1 Mwanza municipality and 4 rural participants.

^9^Defined as waist circumference >94 cm for men and >80 cm for women.

^10^Smoking status results are missing for 1 district town participant.

^11^BMI category results are missing for 5 Entebbe municipality, 3 district town and 1 rural participant.

^12^Waist circumference results are missing for 1 Entebbe municipality and 2 district town participants.

### Population distribution of serum lipids

Table 2 shows the lipid levels and ratios by location and country. Mean TC, LDL-C, HDL-C, apoA1 and apoB levels were highest in municipal areas and lowest in rural populations, with small differences between the countries. LDL-C/HDL-C, apoB/HDL-C, TC/HDL-C and apoB/apoA1 ratios were generally similar across strata in both countries.

**Table 2:** Population distribution of total cholesterol (TC), high-density lipoprotein cholesterol (HDL-C), low-density lipoprotein cholesterol (LDL-C), triglyceride, apolipoprotein A1 (apoA1) and apolipoprotein B (apoB); and apoB/apoA1, LDL-C/HDL-C, apoB/HDL-C and TC/HDL-C ratios.

		Weighted mean^1^ (95% CI)^2^		Weighted mean^1^ (95% CI)^2^		Weighted mean^1^ (95% CI)^2^		P-value^5^	
**TANZANIA**		**Mwanza municipality (n = 170)**		**District towns (n = 326)**		**Rural areas (n = 547)**			
TC (mmol/L)		4.72 (4.48–4.96)		4.42 (4.26–4.59)		3.85 (3.69–4.01)		**<0.001**	
LDL-C (mmol/L)		2.76 (2.60–2.91)		2.49 (2.35–2.63)		2.02 (1.91–2.14)		**<0.001**	
apoB (g/L)^3^		0.85 (0.81–0.89)		0.81 (0.77–0.86)		0.74 (0.71–0.77)		**<0.001**	
HDL-C (mmol/L)		1.39 (1.35–1.43)		1.22 (1.18–1.27)		1.01 (0.93–1.09)		**<0.001**	
apoA1 (g/L)^3^		1.58 (1.51–1.65)		1.43 (1.39–1.47)		1.30 (1.d24-1.35)		**<0.001**	
Triglycerides (mmol/L)		1.05 (0.96–1.14)		1.09 (1.02–1.16)		1.17 (1.09–1.25)		0.12	
apoB/apoA1 ratio^3^		0.56 (0.53–0.59)		0.58 (0.55–0.61)		0.59 (0.57–0.61)		0.24	
LDL-C/HDL-C ratio		2.15 (2.03–2.28)		2.20 (2.04–2.36)		2.22 (2.12–2.32)		0.45	
apoB/HDL-C ratio (g/mmol)^3^		0.68 (0.65–0.71)		0.73 (0.67–0.78)		0.91 (0.82–1.01)		**<0.001**	
TC/HDL-C ratio		3.66 (3.50–3.81)		3.87 (3.65–4.09)		4.43 (4.11–4.75)		**<0.001**	
Non HDL cholesterol		3.33 (3.11–3.54)		3.20 (3.03–3.37)		2.84 (2.74–2.93)		**<0.001**	
**UGANDA**		**Entebbe municipality (n = 205)**		**District towns (n = 278)**		**Rural areas (n = 431)**			
TC (mmol/L)		4.57 (4.40–4.73)		4.35 (4.21–4.49)		4.05 (3.82–4.27)		**<0.001**	
LDL- C (mmol/L)		2.62 (2.48–2.75)		2.50 (2.38–2.62)		2.24 (2.05–2.43)		**0.001**	
apoB (g/L)^4^		0.91 (0.84–0.98)		0.89 (0.85–0.93)		0.84 (0.78–0.91)		0.06	
HDL-C (mmol/L)		1.41 (1.32–1.50)		1.28 (1.23–1.32)		1.16 (1.09–1.23)		**<0.001**	
apoA1 (g/L)^4^		1.49 (1.38–1.61)		1.41 (1.36–1.46)		1.41 (1.35–1.46)		0.22	
Triglycerides (mmol/L)		1.27 (1.02–1.52)		1.27 (1.12–1.42)		1.24 (1.15–1.34)		0.79	
apoB/apoA1 ratio^4^		0.67 (0.57–0.76)		0.67 (0.62–0.73)		0.63 (0.57–0.69)		0.46	
LDL-C/HDL-C ratio		2.04 (1.88–2.20)		2.10 (1.99–2.20)		2.14 (2.00–2.29)		0.72	
apoB/HDL-C ratio (g/mmol)^4^		0.74 (0.65–0.83)		0.77 (0.71–0.83)		0.83 (0.76–0.91)		0.33	
TC/HDL-C ratio		3.53 (3.35–3.72)		3.63 (3.48–3.78)		3.86 (3.63–4.09)		0.10	
Non HDL cholesterol		3.15 (3.00–3.31)		3.08 (2.95–3.21)		2.89 (2.71–3.07)		**0.01**	

^1^Weighted estimates adjusted for survey design with sampling weights applied because the probability of being sampled differed within the strata.

^2^95% confidence interval.

^3^apoA1 and apoB (and hence their ratios) results are missing for 1 rural participant.

^4^apoA1 and apoB (and hence their ratios) results are missing for 1 Entebbe municipality, 4 district town and 14 rural participants.

^5^p-values were calculated using unweighted data by linear regression (Wald test) with each lipid level as the outcome and location as a categorical variable.

### Prevalence of dyslipidemia

Prevalence of dyslipidemia is presented in Table 3. The overall prevalence of dyslipidemia in Tanzania was 30% in Mwanza municipality, 29% in district towns and 50% in rural areas, and in Uganda was 32% in both Entebbe municipality and district towns, and 44% in rural areas. Low HDL-C was the most common dyslipidemia, particularly in rural populations (Tanzania 45%; Uganda 32%). The proportion of the population with high LDL-C was highest in municipal areas (Mwanza 9%; Entebbe 5%) and lowest in rural populations (rural Tanzania 1%; rural Uganda 3%). The prevalence of high apoB was also highest in municipal areas (Mwanza 10%; Entebbe 15%).

**Table 3:** Prevalence of dyslipidaemia.

	Weighted %^1^ (95% CI)^2^	n^3^	Weighted %^1^ (95% CI)^2^	n^3^	Weighted %^1^ (95% CI)^2^	n^3^	P-value^4^
**TANZANIA**	**Mwanza municipality (n = 170)**		**District towns (n = 326)**		**Rural areas (n = 547)**		
High TC (≥6.2 mmol/L)	14.4% (9.1–22.0)	24	5.6% (3.6–8.5)	19	2.6% (1.5–4.5)	13	**<0.001**
High LDL-C (≥4.1 mmol/L)	9.2% (5.3–15.3)	15	4.1% (2.6–6.4)	14	0.9% (0.4–2.1)	5	**<0.001**
High apoB^5^	9.8% (5.8–16.0)	16	7.8% (4.5–13.3)	25	4.8% (3.2–6.9)	29	0.22
Low HDL-C (<1 mmol/L)	15.3% (12.1–19.0)	26	20.6% (15.4–26.9)	67	44.7%(35.9–53.8)	250	**<0.001**
High triglycerides (≥2.26 mmol/L)	3.6% (2.1–6.3)	6	6.3% (4.6–8.6)	21	5.9% (3.5–9.9)	30	0.21
High TC/HDL-C ratio (males >6.4 and females >5.6)	8.1% (5.1–12.7)	14	8.7% (5.1–14.3)	28	13.1% (10.1–16.7)	74	0.07
Any dyslipidaemia ^6^^,^^7^	29.7% (25.7–34.1)	50	29.1% (23.2–35.7)	94	50.0% (41.4–58.7)	278	**<0.001**
Dyslipidaemia other than low HDL^6^	16.9% (12.0–23.4)	28	13.1% (9.1–18.5)	42	10.4% (7.2–14.8)	57	0.21
**UGANDA**	**Entebbe municipality (n = 205)**		**District towns (n = 278)**		**Rural areas (n = 431)**		
High TC (≥6.2 mmol/L)	6.3% (4.4–9.1)	13	5.2% (2.7–9.8)	15	3.6% (1.6–7.6)	13	0.20
High LDL-C (≥4.1 mmol/L)	5.4% (3.3–8.6)	11	3.8% (1.4–9.6)	9	2.8% (1.2–6.3)	10	0.17
High apoB ^8^	14.7% (9.3–22.4)	30	10.7% (7.2–15.5)	34	11.9% (7.5–18.3)	41	0.42
Low HDL-C (<1 mmol/L)	12.7% (9.3–17.1)	26	16.4% (11.2–23.3)	51	31.5% (23.6–40.6)	147	**<0.001**
High triglycerides (≥2.26 mmol/L)	6.8% (4.6–10.1)	14	10.7% (7.7–14.8)	24	9.6% (6.2–14.5)	38	0.56
High TC/HDL-C ratio (males >6.4 and females >5.6)	3.4% (2.0–5.8)	7	4.1% (2.2–7.5)	16	9.7% (6.8–13.6)	42	**0.01**
Any dyslipidaemia ^7^,^9^	31.7% (24.9–39.3)	65	31.5% (25.0–38.9)	90	43.5% (36.5–50.7)	187	**0.05**
Dyslipidaemia other than low HDL^10^	21.0% (15.6–27.6)	43	19.5% (14.9–25.1)	51	18.1% (12.6–25.3)	67	0.48

^1^Weighted estimates, adjusted for survey design with sampling weights applied because the probability of being sampled differed within strata.

^2^95% confidence interval.

^3^Actual number of respondents, without sampling weights applied.

^4^p-values were calculated using unweighted data by logistic regression with location as a categorical variable to compare row variables across the 3 strata.

^5^apoB results are missing for 1 rural participant.

^6^Dyslipidemia results are missing for 1 rural participant.

^7^Dyslipidaemia defined as high TC, LDL-C, triglyceride or apoB, or low HDL-C.

^8^apoB results are missing for 1 Entebbe municipality, 4 district town and 14 rural participants.

^9^Dyslipidemia results are missing for 3 district town and 7 rural participants.

^10^Dyslipidemia other than HDL results are missing for 4 district town and 11 rural participants.

Across all strata, 24% of the population had low HDL-C without any other lipid or apoliprotein abnormalities (S1 Table). All other dyslipidemias commonly occurred in clusters of 2 or 3 abnormalities. LDL-C levels were strongly and positively correlated with apoB levels (Pearson’s correlation coefficient = 0.87); and HDL-C levels were strongly correlated with apoA1 levels (Pearson’s correlation coefficient = 0.79).

### Factors associated with lipid levels

#### Factors associated with high LDL-C and apoB

Table 4 displays factors associated with mean LDL-C and apoB levels. Factors significantly associated with higher mean LDL-C were female gender, older age, higher education, formal employment, higher monthly income, more items owned, tap water in the house compound, never having smoked, higher BMI, abdominal obesity, being HIV negative and having elevated BP levels. Factors significantly associated with higher mean apoB levels were nearly identical to those associated with higher mean LDL-C levels except that formal employment and HIV were not significantly associated with higher mean apoB but using a vehicle as the usual mode of transport was significantly associated with higher mean apoB.

**Table 4:** Factors associated with low-density lipoprotein cholesterol, apolipoprotein B, high-density lipoprotein cholesterol and apolipoprotein A1 levels.

		Factors associated with LDL-C		Factors associated with apoB		Factors associated with HDL-C		Factors associated with apoA1	
	n	Mean	Coefficient (95% CI)^1^	Mean	Coefficient (95% CI)^1^	Mean	Coefficient (95% CI)^1^	Mean	Coefficient (95% CI)^1^
**Fixed variables**									
Sex			**p<0.001**		**p<0.001**		**p<0.001**		**p = 0.007**
Male	852	2.14	**0**	0.76	**0**	1.13	**0**	1.39	**0**
Female	1105	2.45	**0.35 (0.28, 0.43)**	0.86	**0.11 (0.09, 0.14)**	1.22	**0.08 (0.05, 0.11)**	1.43	**0.05 (0.01, 0.08)**
Age, years			**p<0.001**		**p<0.001**		**p = 0.01**		**p<0.001**
<25	554	2.17	**0**	0.76	**0**	1.15	**0**	1.35	**0**
25–34	580	2.30	**0.17 (0.07, 0.27)**	0.81	**0.05 (0.02, 0.09)**	1.20	**0.04 (-0.00, 0.09)**	1.42	**0.05 (0.00, 0.09)**
35–44	322	2.41	**0.32 (0.21, 0.43)**	0.85	**0.10 (0.06, 0.13)**	1.17	**0.04 (-0.02, 0.11)**	1.42	**0.06 (0.00, 0.11)**
45+	501	2.43	**0.48 (0.35, 0.60)**	0.88	**0.16 (0.13, 0.19)**	1.19	**0.11 (0.05, 0.17)**	1.48	**0.14 (0.09, 0.19)**
**Other sociodemographic variables**^**1**^									
Marital status			p = 0.19		p = 0.19		**p = 0.04**		**p = 0.008**
Married/living as married	1167	2.32	0	0.83	0	1.18	**0**	1.43	**0**
Widowed/separated/divorced	344	2.38	-0.06 (-0.16, 0.03)	0.86	-0.00 (-0.04, 0.03)	1.19	**-0.03 (-0.08, 0.02)**	1.44	**-0.03 (-0.07, 0.01)**
Single	446	2.23	-0.08 (-0.20, 0.03)	0.78	-0.03 (-0.07, 0.00)	1.18	**-0.05 (-0.10, -0.01)**	1.35	**-0.06 (-0.09, 0.02)**
Education			**p = 0.01**		**p = 0.01**		**p = 0.007**		p = 0.19
None/incomplete primary	662	2.15	**0**	0.78	**0**	1.11	**0**	1.38	0
Primary	633	2.32	**0.11 (0.01, 0.20)**	0.82	**0.04 (0.01, 0.07)**	1.16	**0.04 (-0.00, 0.09)**	1.42	0.03 (-0.01, 0.08)
Secondary or above	662	2.47	**0.17 (0.05, 0.29)**	0.86	**0.04 (0.00, 0.08)**	1.27	**0.09 (0.03, 0.14)**	1.43	0.04 (-0.01, 0.09)
Employment			**p = 0.01**		p = 0.07		p = 0.81		p = 0.85
Self-employed	1183	2.25	**0**	0.81	0	1.15	0	1.41	0
Formal employment	286	2.56	**0.16 (0.05, 0.28)**	0.88	0.04 (0.01, 0.07)	1.23	0.01 (-0.06, 0.07)	1.43	-0.00 (-0.05, 0.05)
Not employed	488	2.32	**0.01 (-0.10, 0.11)**	0.82	-0.00 (-0.04, 0.03)	1.23	0.02 (-0.04, 0.07)	1.41	0.01 (-0.03, 0.05)
Monthly income, USD			**p = 0.005**		**p = 0.01**		p = 0.57		p = 0.10
<38	1064	2.20	**0**	0.80	**0**	1.15	0	1.39	0
≥38	829	2.48	**0.14 (0.04, 0.23)**	0.86	**0.04 (0.01, 0.07)**	1.23	0.01 (-0.03, 0.06)	1.45	0.03 (-0.01, 0.07)
Number of items owned			**p = 0.002**		**p = 0.001**		p = 0.93		p = 0.99
0–1	219	2.14	**-0.01 (-0.12, 0.09)**	0.78	**0.00 (-0.03, 0.04)**	1.11	-0.01 (-0.07, 0.05)	1.39	-0.00 (-0.05, 0.05)
2–4	1183	2.27	**0**	0.80	**0**	1.18	0	1.41	0
5+	555	2.48	**0.14 (0.06, 0.22)**	0.87	**0.05 (0.02, 0.08)**	1.21	0.00 (-0.04, 0.05)	1.42	-0.00 (-0.04, 0.04)
Water source			**p<0.001**		**p = 0.002**		p = 0.51		p = 0.48
Tap in house/compound	733	2.53	**0**	0.89	**0**	1.28	0	1.47	0
Well	1047	2.12	**-0.20 (-0.33, -0.08)**	0.76	**-0.07 (-0.12, -0.03)**	1.09	-0.01 (-0.08, 0.05)	1.36	-0.02 (-0.07, 0.03)
Other	177	2.60	**0.08 (-0.13, 0.28)**	0.85	**0.01 (-0.05, 0.07)**	1.30	0.05 (-0.07, 0.16)	1.50	0.02 (-0.05, 0.09)
**Behavioural variables**									
Smoking status			**p = 0.006**		**p<0.001**		p = 0.15		p = 0.18
Current	178	2.00	**-0.21 (-0.36, -0.07)**	0.74	**-0.06 (-0.10, -0.03)**	1.21	0.06 (-0.01, 0.13)	1.47	0.04 (-0.01, 0.10)
Ex-smoker	149	2.14	**-0.18 (-0.34, -0.02)**	0.77	**-0.06 (-0.11, -0.02)**	1.18	0.04 (-0.04, 0.12)	1.45	0.04 (-0.03, 0.10)
Never	1629	2.36	**0**	0.83	**0**	1.18	0	1.40	0
Alcohol consumption			p = 0.08		p = 0.24		**p<0.001**		**p<0.001**
Never drinker	1022	2.34	0	0.81	0	1.14	**0**	1.37	**0**
Ex-drinker	451	2.28	-0.03 (-0.13, 0.06)	0.82	-0.01 (-0.04, 0.02)	1.13	**0.01 (-0.03, 0.05)**	1.39	**0.01 (-0.03, 0.04)**
Healthy drinker	336	2.26	-0.13 (-0.23, -0.03)	0.83	-0.03 (-0.07, -0.00)	1.28	**0.11 (0.03, 0.19)**	1.48	**0.09 (0.03, 0.15)**
Unhealthy drinker	146	2.40	-0.07 (-0.21, 0.08)	0.84	-0.01 (-0.05, 0.04)	1.40	**0.23 (0.15, 0.32)**	1.66	**0.26 (0.19, 0.33)**
Usual mode of transport			**p = 0.05**		**p = 0.006**		p = 0.60		p = 0.39
Walk	916	2.28	**0**	0.81	**0**	1.19	0	1.41	0
Bicycle	438	2.09	**-0.06 (-0.17, 0.04)**	0.75	**-0.02 (-0.05, 0.01)**	1.06	-0.01 (-0.06, 0.03)	1.36	-0.02 (-0.06, 0.03)
Vehicle	603	2.54	**0.10 (-0.02, 0.22)**	0.89	**0.05 (0.01, 0.09)**	1.25	-0.02 (-0.07, 0.03)	1.45	-0.02 (-0.06, 0.01)
**Anthropometric variables**									
BMI category			**p<0.001**		**p<0.001**		**p<0.001**		p = 0.10
Underweight	185	1.89	**-0.25 (-0.36, -0.14)**	0.70	**-0.05 (-0.09, -0.02)**	1.16	**0.01 (-0.07, 0.09)**	1.38	0.00 (-0.06, 0.06)
Normal	1309	2.19	**0**	0.78	**0**	1.18	**0**	1.40	0
Overweight	300	2.71	**0.23 (0.11, 0.36)**	0.96	**0.06 (0.01, 0.11)**	1.20	**-0.06 (-0.12, 0.00)**	1.47	-0.00 (-0.05, 0.04)
Obese	149	3.12	**0.48 (0.26, 0.71)**	1.09	**0.15 (0.07, 0.23)**	1.17	**-0.15 (-0.21, -0.09)**	1.45	-0.06 (-0.11, -0.01)
**Abdominal obesity**^**2**^			**p = 0.03**		**p<0.001**		p = 0.10		p = 0.13
No	1443	2.15	**0**	0.76	**0**	1.18	0	1.39	0
Yes	511	2.77	**0.12 (0.01, 0.24)**	0.99	**0.09 (0.05, 0.13)**	1.18	-0.05 (-0.12, 0.01)	1.47	0.04 (-0.01, 0.09)
**Chronic disease variables**									
HIV			**p = 0.01**		p = 0.73		**p<0.001**		**p<0.001**
Negative	1760	2.33	**0**	0.82	0	1.19	**0**	1.42	**0**
Positive and off treatment	125	2.16	**-0.22 (-0.38, -0.06)**	0.82	-0.02 (-0.07, 0.04)	0.96	**-0.29 (-0.37, -0.21)**	1.25	**-0.22 (-0.29, -0.16)**
Positive and on treatment	62	2.20	**-0.12 (-0.33, 0.08)**	0.82	-0.01 (-0.09, 0.06)	1.25	**0.04 (-0.11, 0.18)**	1.44	**0.01 (-0.10, 0.11)**
Hypertension			**p<0.001**		**p = 0.004**		**p = 0.02**		**p<0.001**
Negative	1573	2.24	**0**	0.80	**0**	1.17	**0**	1.39	**0**
Positive and off treatment	355	2.56	**0.23 (0.12, 0.33)**	0.90	**0.06 (0.02, 0.09)**	1.22	**0.06 (0.01, 0.12)**	1.51	**0.08 (0.04, 0.12)**
Positive and on treatment	29	3.21	**0.26 (-0.14, 0.67)**	1.16	**0.08 (-0.07, 0.24)**	1.29	**0.09 (-0.00, 0.18)**	1.47	**0.03 (-0.08, 0.13)**

Note: “0” indicates the reference group. Results of the variables with p≤0.05 in the final models are shown in bold.

^1^95% confidence interval

^2^abdominal obesity was defined as waist circumference >94 cm for men and >80 cm for women.

#### Factors associated with low HDL-C and apoA1

Table 4 also displays factors associated with mean HDL-C and apoA1 levels. Factors significantly associated with lower mean HDL-C levels were female gender, younger age, being single, lower level of education, less alcohol consumption, higher BMI, being HIV-infected (off treatment) and not having hypertension. All of the factors associated with lower mean apoA1 levels were among those significantly associated with lower mean HDL-C levels except lower level of education and BMI category.

### Framingham cardiovascular risk scores

Table 5 displays the Framingham 10-year cardiovascular risk scores calculated with and without lipid values. Categorization of 10-year cardiovascular risk scores was generally similar between the scores with and without lipids, with the proportion of the population classified as intermediate and high risk being within 9–20% in each country and strata. The proportion of the population with high risk scores (without lipids) was generally lower in urban areas (Tanzania 5.6%; Uganda 5.5%) and higher in rural areas (Tanzania 7.1%; Uganda 11.2%).

**Table 5:** Framingham cardiovascular risk scores.

	Weighted %^1^ (95% CI)^2^	n^3^	Weighted %^1^ (95% CI)^2^	n^3^	Weighted %^1^(95% CI)^2^	n^3^	P-value^4^
**TANZANIA**	**Mwanza municipality (n = 170)**		**District towns (n = 326)**		**Rural areas (n = 547)**		
Framingham 10-year cardiovascular risk category (without lipid results)^5^							0.06
Low (<10%)	86.0% (80.2–90.2)	147	89.4% (84.9–92.7)	291	82.6% (77.9–86.4)	446	
Intermediate (10–19%)	8.4% (4.5–15.3)	13	7.1% (4.9–10.2)	23	10.3% (7.6–13.7)	56	
High (≥20%)	5.6% (2.7–11.1)	9	3.5% (2.1–5.9)	12	7.1% (4.9–10.2)	41	
Framingham 10-year cardiovascular risk category (with lipid results)							**0.05**
Low (<10%)	88.1% (81.4–92.6)	151	90.7% (86.2–93.8)	295	84.4% (80.2–87.9)	458	
Intermediate (10–19%)	7.6% (3.7–15.2)	12	5.7% (3.8–8.5)	19	8.8% (6.4–11.9)	50	
High (≥20%)	4.3% (2.2–8.0)	7	3.6% (1.9–6.7)	12	6.8% (4.7–9.7)	39	
**UGANDA**	**Entebbe municipality (n = 205)**		**District towns (n = 278)**		**Rural areas (n = 431)**		
Framingham 10-year cardiovascular risk category (without lipid results)^6^							**0.01**
Low (<10%)	90.0% (84.2–93.8)	180	88.9% (83.4–92.7)	246	80.7% (72.7–86.7)	344	
Intermediate (10–19%)	4.5% (2.3–8.7)	9	4.1% (2.3–7.2)	14	8.2% (5.8–11.4)	34	
High (≥20%)	5.5% (3.0–10.0)	11	7.0% (3.7–12.9)	15	11.2% (6.7–18.0)	52	
Framingham 10-year cardiovascular risk category (with lipid results)							**0.006**
Low (<10%)	91.2% (86.5–94.4)	187	90.4% (86.3–93.4)	254	82.7% (76.1–87.7)	353	
Intermediate (10–19%)	2.9% (1.9–4.4)	6	1.5% (0.6–4.1)	10	7.9% (5.7–10.8)	35	
High (≥20%)	5.9% (3.3–10.1)	12	8.0% (4.7–13.3)	14	9.4% (6.3–14.0)	43	

^1^Weighted estimates, adjusted for survey design with sampling weights applied because the probability of being sampled differed within the strata.

^2^95% confidence interval.

^3^Actual number of respondents, without sampling weights applied.

^4^p-values were calculated using unweighted data by ordinal logistic regression with location as a categorical variable.

^5^Framingham risk scores results are missing for 1 Mwanza municipality and 2 rural participants (missing weight and/or height).

^6^Framingham risk scores results are missing for 5 Entebbe municipality, 3 district town and 1 rural participants (missing weight and/or height).

We also calculated Framingham 10-year cardiovascular risk scores (without lipid values) by age category in the overall study population. Among those aged <35 years, all 1162 participants were categorised as low risk by Framingham. Among those aged 35–44 years, 12/326 (3.7%) and 2/326 (0.6%) were intermediate and high risk, respectively. The corresponding figures among those aged 45–55 years were 63/237 (26.6%) and 13/237 (5.5%); and among 55+ years were 80/267 (30.0%) and 126/267 (47.2%). Of note, in the 55+ years group, 72/267 participants (30.0%) had 10-year cardiovascular risk scores of >30% compared to only 4 individuals in all other groups combined.

## Discussion

Our study is the first multi-national, community-based survey of dyslipidemia and cardiovascular risk scores in Africa. The distribution of dyslipidemias and cardiovascular risk was similar between Uganda and Tanzania and differed between rural and urban areas in both countries. We found that 30–50% of the population in the study areas meet the internationally-recognized Adult Treatment Panel III criteria for dyslipidemia but only few (<10%) have high 10-year cardiovascular risk scores. The relatively higher prevalence of dyslipidemia found in our study compared to prior studies is probably because we tested for a broad spectrum of dyslipidemias compared to prior studies [7, 31, 32].

Elevated levels of LDL-C were rare in our study population, occurring in <10% of population all study strata. Similarly low LDL-C levels have been reported in African Americans living in the United States [33], suggesting a possible genetic protection. However, LDL-C levels were relatively higher in urban than rural areas, as previously described in West Africa [11, 33]. Female gender, older age and obesity were all consistently associated with higher LDL-C and apoB levels, as previously reported in ecological studies [4]. The observed associations could partly be explained by genetic predisposition such *GYP19A1 rs10046* polymorphism. This gene variant has been associated with high apoB levels among non-obese females and obese men [34]. Association of LDL-C and apoB with older age may reflect cumulative effect of exposure to predisposing risk factors to high blood lipid levels including physical inactivity [35] and dietary habits [36]. Reducing LDL-C levels with HMG Co-A reductase inhibitors (i.e. statins) has consistently been shown to reduce both cardiovascular and overall mortality risk [37], but very few Africans in need of treatment are currently receiving it [31, 38]. Health services should be strengthened to include dyslipidemia management, particularly in urban areas and for older adults with obesity [39, 40].

Low HDL-C was the most common dyslipidemia, occurring in 30–40% of the rural population. Unlike other dyslipidemias, low HDL-C levels often occurred alone (without other associated dyslipidemias). Although a high prevalence of low HDL-C has previously been reported in rural communities of other African countries [32, 41], the clinical importance of this abnormality remains uncertain. In North American and European populations, adults with low HDL-C have a higher incidence of coronary heart disease [42]. No prospective data has been published from Africa. The fact that lower HDL-C levels correlated with lower LDL-C levels in our population suggests that production of HDL-C is likely low because of the low need for cholesteryl ester reverse transport. This hypothesis is supported by the relatively high apoA1 levels in our study population compared to prior studies in Africa [1, 38]. Alternatively, low HDL-C and apoA1 levels may be related to insufficient macronutrient or micronutrient (particularly B12 or zinc) intake [13, 43]. Further research is warranted.

We also reported Framingham 10-year cardiovascular risk scores using 2 formulae: with and without lipids. These formulae yielded similar results in all strata, supporting the notion that simple clinical risk scores without lipid measurement could be sufficient for identifying those with high cardiovascular risk in resource-limited countries in Africa [44]. Nearly all adults with high cardiovascular risk scores were older (>55 years of age). Therefore, screening for cardiovascular risk in Africa should begin with this group. Identifying adults with high cardiovascular risk is clinically important because lipid-lowering drugs have been shown to be cost-effective in this group [45].

Our study has limitations. Although we used 10-year Framingham risk equation in this study, it has not been validated prospectively in African populations. Development and validation of a region-specific risk estimation equation is needed.

In conclusion, dyslipidemias are common in both urban and rural populations in Tanzania and Uganda. Low HDL-C was the most commonly observed dyslipidemia, affecting nearly one third of rural populations. Elevated LDL-C was relatively rare but was more prevalent in older, overweight, urban dwelling females of higher socioeconomic status. Although population cardiovascular risk scores both with and without lipids were generally low, many adults >55years had high cardiovascular risk scores. Health services designed and equipped to diagnose and treat dyslipidemia are urgently needed.

## Supporting information

S1 Table Individual and combined dyslipidemias in 1957 total study subjects. (DOCX)

S1 Appendix Dataset. (XLS)

S2 Appendix Data dictionary. (XLS)
