# Supplementary material for: Dyslipidemias and cardiovascular risk scores in urban and rural populations in north-western Tanzania and southern Uganda
Source: PLoS One. 2019 Dec 6;14(12):e0223189. doi: 10.1371/journal.pone.0223189 (PMC6897412; doi:10.1371/journal.pone.0223189)
Supplement: S1 Table — (DOCX) [file pone.0223189.s001.docx]

**S1 Table. Individual and combined dyslipidemias in 1957 total study subjects**

| **Category** | **Unweighted N (%)** |
| --- | --- |
| All normal [1] | 1193 (61%) |
| **1 abnormality** | |
| Low HDL only [2] | 476 (24%) |
| High apoB only | 58 (3%) |
| High triglycerides only [3] | 35 (2%) |
| High TC only | 16 (1%) |
| High LDL only | 2 (<1%) |
| **2 abnormalities** | |
| Low HDL and high triglycerides [4] | 46 (2%) |
| Low HDL and high apoB | 22 (1%) |
| High triglycerides and apoB | 16 (1%) |
| High TC and triglycerides | 6 (<1%) |
| High TC and apoB | 6 (<1%) |
| High LDL and apoB | 4 (<1%) |
| High TC and LDL | 3 (<1%) |
| **3 abnormalities** | |
| High TC, LDL and apoB | 37 (2%) |
| High TC, triglycerides and apoB | 7 (<1%) |
| Low HDL and high triglycerides and high apoB | 6 (<1%) |
| High TC and high triglycerides and low HDL | 4 (<1%) |
| High LDL, high triglycerides and high apoB | 1 (<1%) |
| Low HDL and high LDL and apoB | 1 (<1%) |
| **4 or more abnormalities** | |
| High TC, LDL, triglycerides and apoB | 6 (<1%) |
| High TC, LDL and apoB and low HDL | 5 (<1%) |
| High TC, LDL, triglycerides, apoB and low HDL | 4 (<1%) |
| High TC, triglycerides, apoB and low HDL | 2 (<1%) |
| High TC, LDL and triglycerides and low HDL | 1 (<1%) |

[1] Includes 11 participants with apoB results missing. [2] Includes 5 participants with apoB results missing. [3] Includes 1 participant with apoB results missing. [4] Includes 3 participants with apoB results missing.
